# The anti-HER3 (ErbB3) therapeutic antibody 9F7-F11 induces HER3 ubiquitination and degradation in tumors through JNK1/2- dependent ITCH/AIP4 activation

**DOI:** 10.18632/oncotarget.9455

**Published:** 2016-05-18

**Authors:** Christophe Le Clorennec, Yassamine Lazrek, Olivier Dubreuil, Christel Larbouret, Marie-Alix Poul, Philippe Mondon, Gerry Melino, André Pèlegrin, Thierry Chardès

**Affiliations:** ^1^ IRCM, Institut de Recherche en Cancérologie de Montpellier, Montpellier, F-34298, France; ^2^ INSERM, U1194 Montpellier, Montpellier, F-34298, France; ^3^ Université de Montpellier, Montpellier, F-34298, France; ^4^ ICM, Institut Régional du Cancer Montpellier, Montpellier, F-34298, France; ^5^ Millegen SA, Labège, F-31670, France; ^6^ Biochemistry Laboratory, Instituto Dermopatico Dell'Immacolata, Department of Experimental Medicine and Surgery, University of Rome “Tor Vergata,” 00133 Rome, Italy; ^7^ Toxicology Unit, Medical Research Council, Leicester University, Leicester LE1 9HN, United Kingdom; ^8^ Institut Pasteur de Guyane, BP 6010, 97306, Cayenne Cedex, France; ^9^ GamaMabs Pharma SA, Centre Pierre Potier, ONCOPOLE, BP 50624, France; ^10^ LFB Biotechnologies, 59000, Lille, France

**Keywords:** cancer, HER3, ITCH/AIP4, antibody, treatment

## Abstract

We characterized the mechanism of action of the neuregulin-non-competitive anti-HER3 therapeutic antibody 9F7-F11 that blocks the PI3K/AKT pathway, leading to cell cycle arrest and apoptosis *in vitro* and regression of pancreatic and breast cancer *in vivo*. We found that 9F7-F11 induces rapid HER3 down-regulation. Specifically, 9F7-F11-induced HER3 ubiquitination and degradation in pancreatic, breast and prostate cancer cell lines was driven mainly by the itchy E3 ubiquitin ligase (ITCH/AIP4). Overexpression of the ITCH/AIP4 inhibitor N4BP1 or small-interfering RNA-mediated knockdown of ITCH/AIP4 inhibited HER3 ubiquitination/degradation and PI3K/AKT signaling blockade induced by 9F7-F11. Moreover, 9F7-F11-mediated JNK1/2 phosphorylation led to ITCH/AIP4 activation and recruitment to HER3 for receptor ubiquitination and degradation. ITCH/AIP4 activity was activated by the deubiquitinases USP8 and USP9X, as demonstrated by RNA interference. Taken together, our results suggest that 9F7-F11-induced HER3 ubiquitination and degradation in cancer cells mainly occurs through JNK1/2-dependent ITCH/AIP4 activation.

## INTRODUCTION

HER3/ErbB3 is a member of the human epidermal growth factor receptor (EGFR) family [1] and HER3 expression has been correlated with tumor progression and reduction of patient survival in pancreatic, breast, ovarian and gastric cancer, in head and neck squamous cell carcinoma and in melanoma [2–7]. Therefore, much effort is currently focused on the development of anti-HER3 therapeutic antibodies for cancer treatment [8, 9]. Most of these molecules block the NRG-1 binding site [10–12]; however, HER3 can act through ligand-independent and -dependent oncogenic mechanisms [13] *via* paracrine or autocrine loops [14]. This allowed us to generate the neuregulin-non-competitive anti-HER3 antibody 9F7-F11 that blocks the PI3K/AKT pathway, leading to *in vivo* tumor regression [15]. Interestingly, a strong HER3 down-regulation is observed *in vitro* in tumor cells co-incubated with 9F7-F11 and NRG-1 [15]. Here, we investigated the molecular mechanisms of HER3 degradation induced by 9F7-F11 to better understand the anti-cancer efficiency of this therapeutic antibody.

Proteasomal or lysosomal degradation of tyrosine kinase receptors, and particularly of EGFR family receptors, is regulated by ubiquitination [16, 17]. E3 ubiquitin ligases have crucial roles in regulating EGFR family receptor levels [18]. The RING-finger E3 ubiquitin ligase CBL binds to EGFR and favors its ubiquitination for lysosomal degradation [19], whereas the E3 ubiquitin ligase CHIP promotes HER2 ubiquitination/degradation [20]. Other HECT family E3 ligases, such as WW domain containing protein 1 (WWP1) and itchy E3 ubiquitin protein ligase (ITCH/AIP4), ubiquitinate HER4, leading to its proteasomal and lysosomal degradation [21, 22]. Dysregulation of EGFR ubiquitination/degradation has been related to resistance to cetuximab [23]. The therapeutic efficiency of anti-EGFR, -HER2 and -c-MET antibodies, used alone or in combination, is associated with an increase of receptor ubiquitination and degradation in tumor cells [24–30], sometimes mediated by the E3 ubiquitin ligase CBL [28, 29]. The RING-finger E3 ubiquitin ligase neuregulin receptor degradation protein-1 (Nrdp1) and the HECT family E3 neural precursor cell expressed, developmentally downregulated 4 (NEDD4) ubiquitinate HER3 in basal condition and after NRG-1 stimulation [31–33], but nothing is known about the mechanisms of drug-induced HER3 degradation/ubiquitination.

Here, we show that the E3 ubiquitin ligase ITCH/AIP4 binds to HER3 and activates HER3 ubiquitination and degradation induced by 9F7-F11. ITCH knock-down by small interfering RNA abrogated 9F7-F11-induced HER3 ubiquitination and proteasomal degradation in pancreatic, prostate and breast cancer cells. 9F7- F11-mediated ITCH activation occurred in a JNK1/2-dependent manner and was activated by the ITCH-specific deubiquitinases USP8 and USP9X. Collectively, these data demonstrate that the anti-HER3 therapeutic antibody 9F7- F11 promotes HER3 degradation to inhibit the progression of HER3-dependent tumors.

## RESULTS

### The anti-HER3 antibody 9F7-F11 induces HER3 degradation both in NRG-1β-stimulated and non-stimulated pancreatic, prostate and breast cancer cells

**Figure 1:**
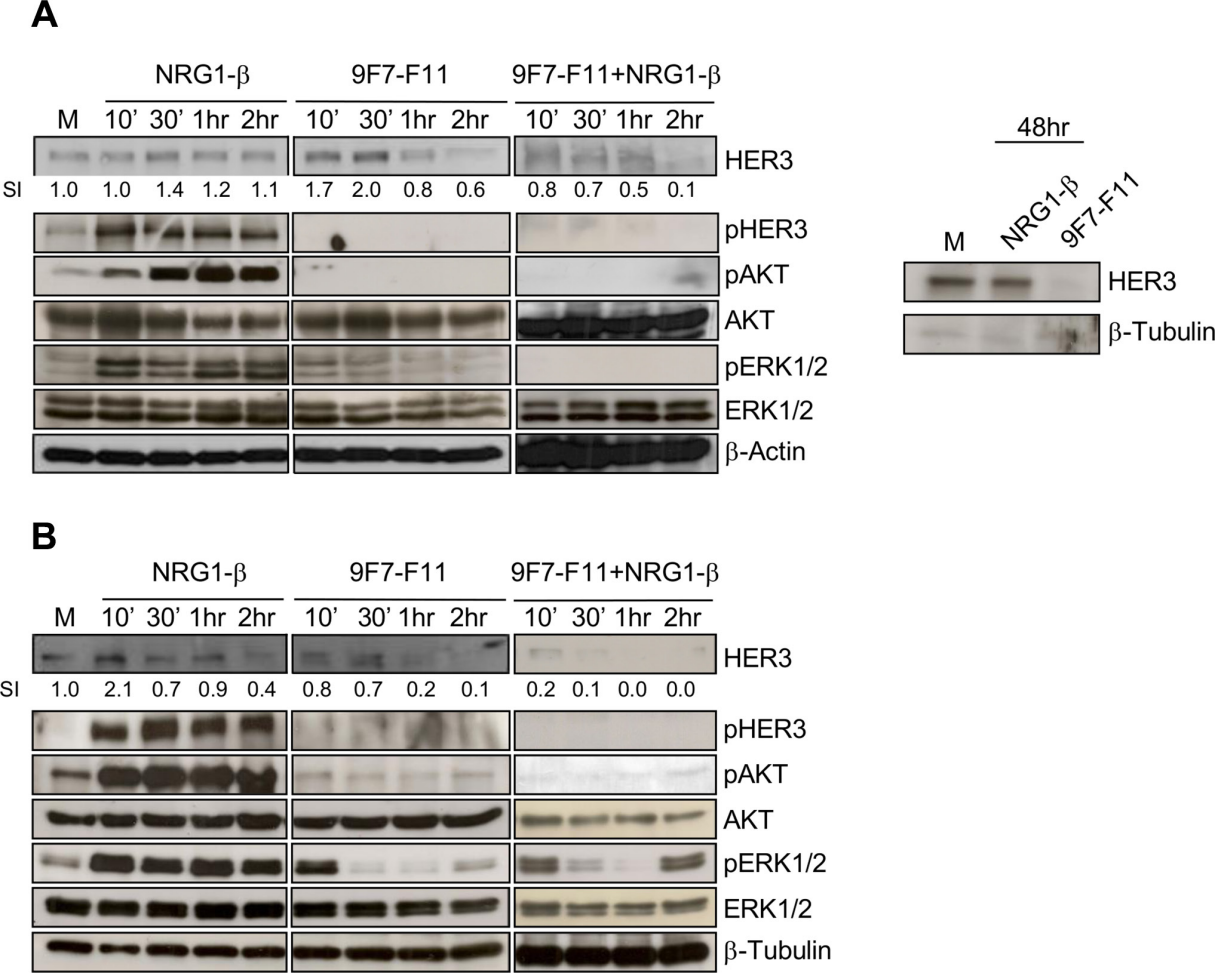
The anti-HER3 antibody 9F7-F11 inhibits NRG1-β-induced HER3 activation, leading to the blockade of the AKT and ERK pathways, and to HER3 degradation in cancer cells Pancreatic BxPC3 (**A**) and prostatic DU145 (**B**) cancer cells were starved in 1% serum for 24 hr before incubation with 100 ng/mL NRG-1β or/and with 50 μg/mL of the anti-HER3 antibody 9F7- F11 for the indicated times. Quantification of the HER3 signal intensity (SI) with ImageJ is indicated below the images (relative to control). β-actin was evaluated as loading control.

To temporally characterize 9F7-F11-induced HER3 degradation, we incubated pancreatic BxPC3 and prostate DU145 cancer cells with 9F7-F11 in the presence or not of NRG-1β. In BxPC3 cells, 1 to 2 hr-incubation with 9F7-F11 reduced HER3 expression by 20 and 40%, respectively, compared to control cells (medium alone) (Figure 1A). The antibody effect was earlier (after 10 min incubation) and stronger (up to 90% reduction of HER3 expression after 2 hr) in DU145 prostate cells (Figure 1B). After 48 hr, HER3 re-expression was not observed (Figure 1A) in BxPC3 cells, suggesting that 9F7- F11 has a sustained effect on HER3 degradation and does not induce transcription to maintain HER3 expression, as observed following stimulation with NRG-1β [34]. In contrast, treatment of BxPC3 cells with irrelevant control antibody Px did modify neither HER3 expression and phosphorylation, nor subsequent downstream cell signaling (Supplementary Figure S1). In cells co-incubated with NRG-1β, 9F7-F11 effect on HER3 degradation was stronger. HER3 expression was almost completely abrogated in BxPC3 cells after 2 hr of co-incubation (Figure 1A) and in DU145 cells after only 30 min (Figure 1B); such antibody-induced HER3 degradation being confirmed in MDA-MB468 triple-negative breast cancer (Supplementary Figure S2). NRG1-β stimulation alone did not induce HER3 down-regulation in BxPC3 cells during the 2 hr of the experiment (Figure 1A). Conversely, HER3 expression was progressively reduced in DU145 cells after 2 hr-stimulation with NRG1-β, albeit to a lower extent than with 9F7-F11 (Figure 1B), probably due to faster HER3 turnover. Incubation with 9F7-F11 also inhibited phosphorylation of HER3, AKT and ERK1/2 (Figure 1A and 1B). This effect was stronger in cells co- incubated with NRG-1β. A lower efficiency of antibody 9F7-F11 on pERK1/2 was observed in DU145 cells, probably due to the fact that this prostate cancer cell line exhibits KRAS mutation G12V [35]. Conversely, NRG- 1β stimulation alone promoted HER3, AKT and ERK1/2 phosphorylation.

### 9F7-F11-induced HER3 degradation occurs mainly through the proteasome pathway

**Figure 2:**
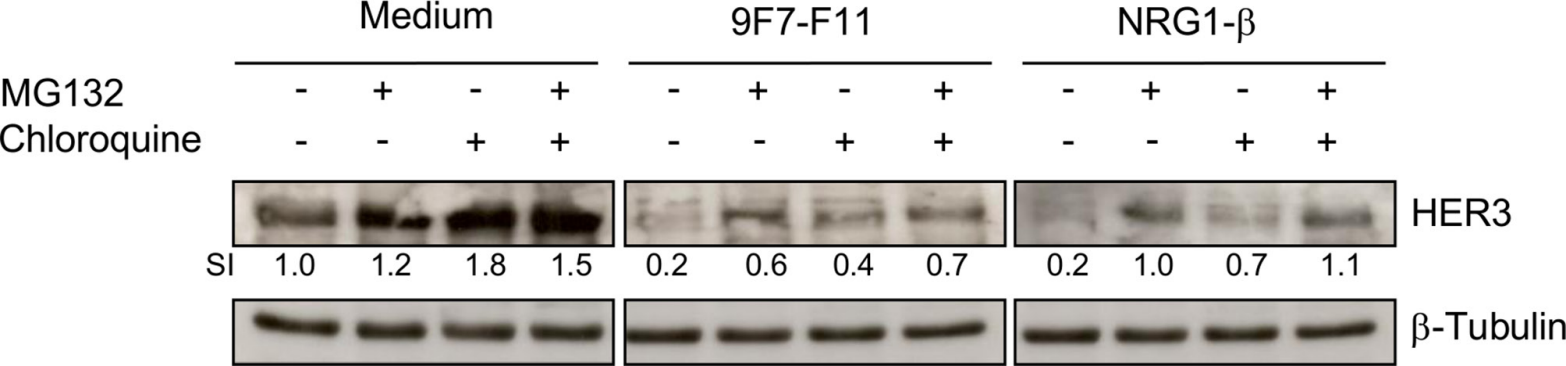
9F7-F11-induced HER3 degradation mainly occurs through the proteasome pathway Serum-starved BxPC3 cells were pre-incubated with 10 μM of the proteasome inhibitor MG132 and/or with 100 μM of the lysosome inhibitor chloroquine for 4 hr, before addition of 50 μg/mL 9F7-F11 or 100 ng/mL NRG-1β for 4 hr. After cell lysis, HER3 protein expression was assessed in whole cell lysates by western blotting using the anti-HER3 antibody C-17. β-tubulin was evaluated as loading control. HER3 protein level was measured with the ImageJ software and indicated as Signal Intensity (SI) relative to control.

To determine the pathways involved in 9F7-F11-induced HER3 degradation, we co-incubated BxPC3 cells for 4 hr with 9F7-F11, NRG-1β or medium alone after pre-incubation with the proteasome inhibitor MG132, the lysosome inhibitor chloroquine or both. Without antibody treatment (medium alone), and consistent with previous studies [32], 4 hr-incubation with chloroquine (SI = 1.8) and, to a lower extent, with MG132 (SI = 1.2), led to HER3 accumulation (Figure 2). 9F7-F11-induced HER3 decrease (SI = 0.2) was substantially reduced by pre-incubation with MG132 (SI = 0.6) and, to a lower extent, with chloroquine (0.4). Combination of the two inhibitors restored HER3 expression (Figure 2). This suggests that 9F7-F11-induced degradation mainly occurs through the proteasome pathway, with a minor contribution from the lysosome pathway. NRG-1β-induced HER3 degradation was mainly via the proteasome pathway (Figure 2), as previously shown upon 3 hr-ligand activation [32].

### The RING E3 ubiquitin ligase Nrdp1 is involved in NRG-1β-mediated, but not in 9F7-F11-induced HER3 degradation in BxPC3 cells

**Figure 3:**
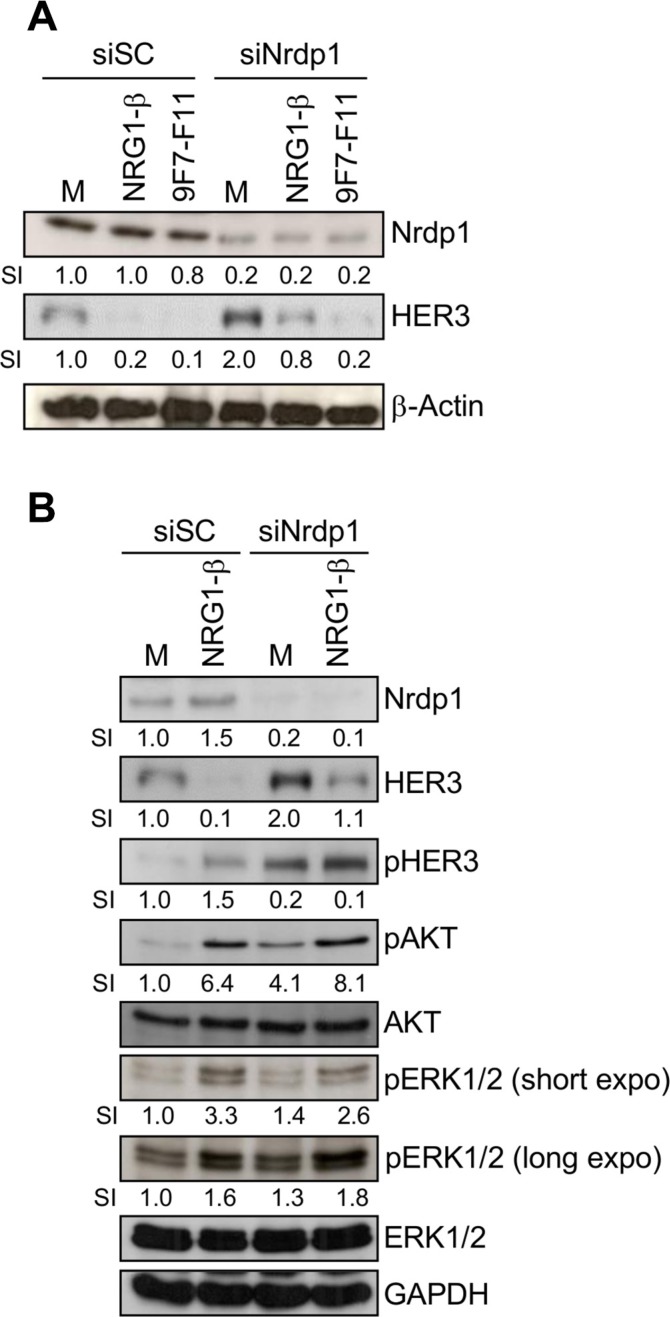
The E3 ubiquitin ligase Nrdp1 is involved in NRG1-β-induced, but not in 9F7-F11-induced HER3 degradation in pancreatic BxPC3 cells (**A**) Cells were transfected with 50 nM of the anti-*Nrdp1* siRNA (siNrdp1) or Scramble Control (siSC) for 72 hr. After starvation, cells were then incubated with 50 μg/mL 9F7-F11 or 100 ng/mL NRG-1β for 4 hr. After cell lysis, HER3 and Nrdp1 expression were assessed in whole protein extracts by western blotting. The signal intensity (SI) (relative to control) was quantified with ImageJ and β-actin was used as loading control. (**B**) Cells transfected with siSC or siNrdp1 were stimulated with NRG-1β for 4 hr. HER3 and Nrdp1 expression as well as HER3, AKT and ERK1/2 phosphorylation were assessed in whole protein extracts by western blotting. GAPDH was used as loading control.

HER3 can be ubiquitinated by Nrdp1 for proteasomal degradation, both in basal conditions and after NRG- 1β stimulation [32, 36]. To determine whether Nrdp1 is involved in 9F7-F11-induced HER3 degradation, we knocked-down the *Nrdp1* gene by transfecting BxPC3 cells with a specific siRNA (siNrdp1) before incubation with 9F7-F11 or NRG-1β for 4 hr. Nrdp1 protein level was reduced by 80% in *Nrdp1*-silenced BxPC3 cells compared to BxPC3 cells transfected with scramble siRNA (siSC) in all experimental conditions (Figure 3A). When cells were incubated with medium alone, *Nrdp1* silencing increased HER3 protein level by 2-fold (Figure 3A), and also AKT and ERK1/2 phosphorylation (Figure 3B), compared to siSC-transfected cells. Treatment of BxPC3 cells with control antibody Px did not modify Nrdp1 expression (Supplementary Figure S1). As expected [36], *Nrdp1* silencing blocked NRG-1β-induced HER3 degradation induced after long term-ligand incubation (4 hr), compared to siSC (Figure 3A and 3B), confirming that Nrdp1 is involved in NRG-1β-mediated HER3 degradation. Conversely, *Nrdp1* silencing did not affect 9F7-F11-induced HER3 degradation (Figure 3A).

### The anti-HER3 antibody 9F7-F11 induces recruitment of the E3 ubiquitin ligase ITCH for HER3 ubiquitination

**Figure 4:**
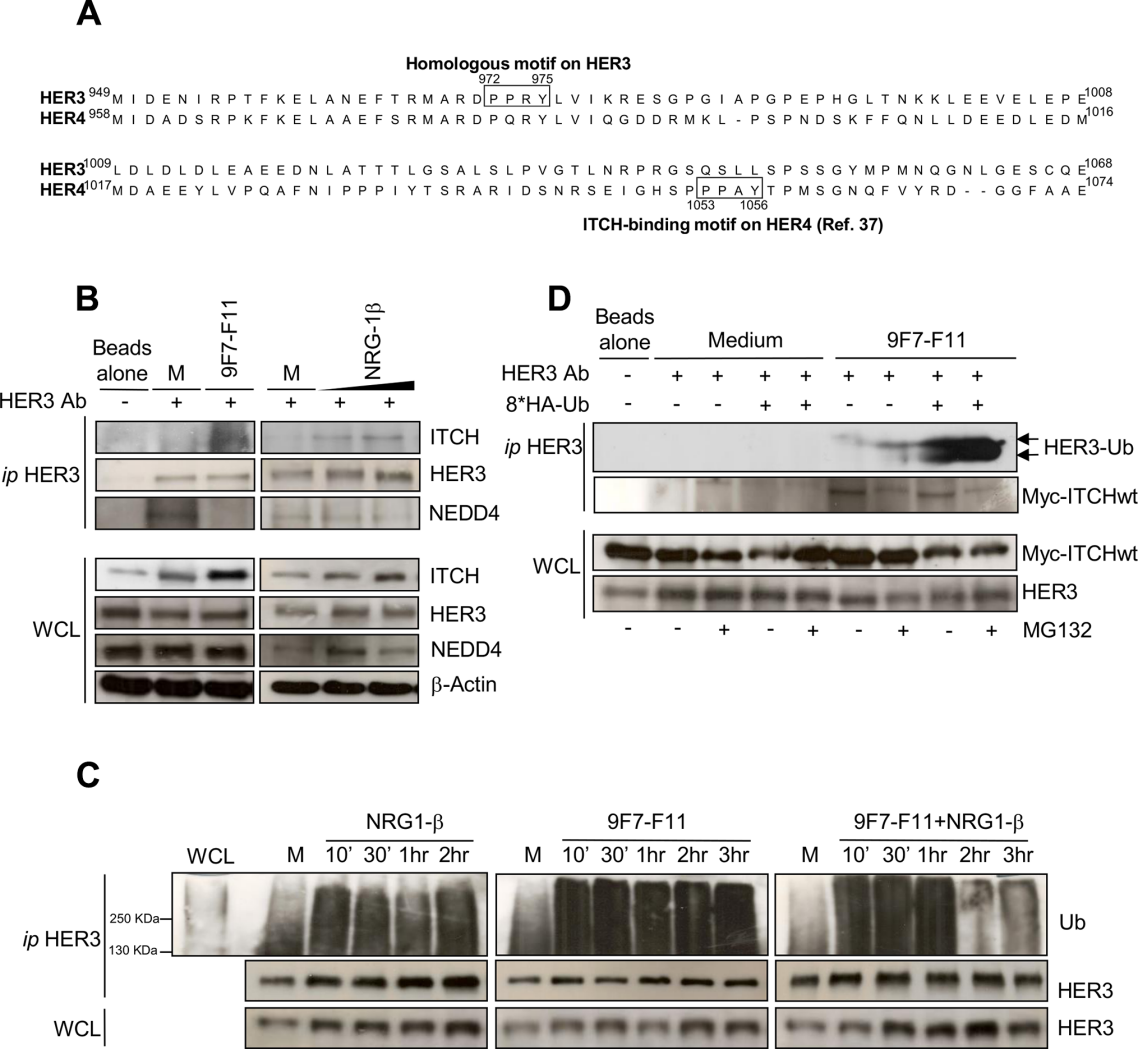
ITCH interacts with HER3 and the anti-HER3 antibody 9F7-F11 induces ITCH recruitment leading to HER3 ubiquitination and disruption of NEDD4/HER3 interaction (**A**) Protein sequence alignment of HER3 and HER4 C-terminal tails showing the homology between the HER3 ^972^PPRY^975^ motif and the ITCH-binding motif ^1053^PPAY^1056^in HER4. (**B**) Serum-starved BxPC3 cells were incubated with 50 μg/mL 9F7-F11 for 30 min or with 100ng/mL NRG-1β for 30 min or 1 hr. After immunoprecipitation (ip) of 2 mg of total protein extracts with an anti-HER3 monoclonal antibody against HER3 C-terminal tail (HER3 Ab), ITCH, HER3 and NEDD4 were detected by western blotting. (**C**) Serum-starved BxPC3 cells were pre-incubated with 10 μM MG132 for 4 hr before incubation with 50 μg/mL 9F7-F11 with or without 100 ng/mL NRG-1β for the indicated times. After immunoprecipitation with HER3 Ab, HER3 ubiquitination status was assessed by western blotting using a specific anti-ubiquitin antibody (Ub). (**D**) MDA-MB468 cells were transfected with the Myc-ITCH plasmid and the HA-Ubiquitin plasmid or not for 24 hr. Then, cells were incubated with 20 μM MG132 for 5 hr before addition of 50 μg/mL 9F7-F11 for 2 hr. After immunoprecipitation with HER3 Ab, HER3 was probed with an ubiquitin-specific antibody as in (C). WCL, whole cell lysate.

The E3 ubiquitin ligases ITCH/AIP4, NEDD4 and WWP1 from the HECT family decrease HER4 protein level [21, 22, 37, 38]. ITCH/AIP4 binds directly to the ^1153^PPX(A)Y^1156^ motif in the CYT1 domain of HER4 [37]. HER3 and HER4 protein sequence alignment revealed a PPX(R)Y binding motif (amino acids 972 to 975) in HER3 C-terminal tail (Figure 4A) that could potentially be involved in HER3/ITCH interaction.

To assess whether HER3 binds to ITCH, we immunoprecipitated with a C-terminal tail-specific anti-HER3 antibody (HER3 Ab) protein extracts from BxPC3 cells (Figure 4B and Supplementary Figure S3) following pre-incubation with medium alone (control), 9F7-F11 or NRG-1β. In the absence of HER3 Ab for immunoprecipitation (Figure 4B) no HER3 detection was observed in the immunoprecipitation extract whereas HER3 was evidenced in the whole cell lysate. When HER3 Ab was added, ITCH was co-immunoprecipitated with HER3 only in cells incubated with 9F7-F11 or NRG- 1β. The absence of ITCH recruitment in control cells treated with medium alone suggests that ITCH needs to be activated to bind to its substrates, as previously demonstrated in HEK293 cells [39]. Normal control antibody did immunoprecipitate neither HER3 nor ITCH (Supplementary Figure S3). The same HER3 motif was recently identified as the NEDD4 binding site for HER3 ubiquitination and degradation, independently of NRG-1β stimulation [33]. Therefore, we used an anti-NEDD4 antibody to check whether NEDD4 was co- immunoprecipitated with HER3 in BxPC3 cells (Figure 4B). Differently from ITCH, NEDD4 co- immunoprecipitated with HER3 only in basal conditions (medium alone), but not after incubation with 9F7-F11 or with NRG-1β, as previously shown [33]. 9F7-F11-induced abrogation of NEDD4/HER3 interaction could be due to competition between ITCH and NEDD4 for the same binding site on HER3, with ITCH replacing NEDD4 upon 9F7-F11 treatment. Taken together, these results show that 9F7-F11 induces the direct recruitment of ITCH on HER3 to drive HER3 degradation in BxPC3 cells.

As ITCH ubiquitinates receptors of the EGFR family [37], we asked whether 9F7-F11-induced ITCH recruitment could lead to HER3 ubiquitination. We immunoprecipitated with an anti-HER3 antibody extracts of BxPC3 cells pre-incubated with MG132 and then incubated with medium alone (control), 9F7-F11 or/and NRG-1β (Figure 4C). Analysis of HER3 ubiquitination in the immunoprecipitates showed that HER3 ubiquitination increased upon incubation with 9F7-F11, with or without NRG-1β, compared to control. HER3 ubiquitination was also observed, but at a lower level, in medium-treated cells; this effect being probably due to NEDD4-mediated ubiquitination at steady state [33]. Reduction of HER3 ubiquitination following 2–3 hr-treatment with antibody plus NRG-1β could be due to ligand-mediated ITCH auto-ubiquitination and further degradation [40]. Antibody-induced HER3 ubiquitination was confirmed in MDA- MB468 breast cancer cells (Supplementary Figure S4). In MDA-MB468 cells transiently transfected with plasmids expressing wild type Myc-ITCH and HA-ubiquitin, incubation with 9F7-F11 induced HER3 ubiquitination (Figure 4D). Taken together, these results show that the anti-HER3 antibody 9F7-F11 induces ITCH recruitment for HER3 ubiquitination and degradation.

### Overexpression of the ITCH inhibitor N4BP1 inhibits 9F7-F11-induced HER3 ubiquitination and degradation and stabilizes HER3 expression

**Figure 5:**
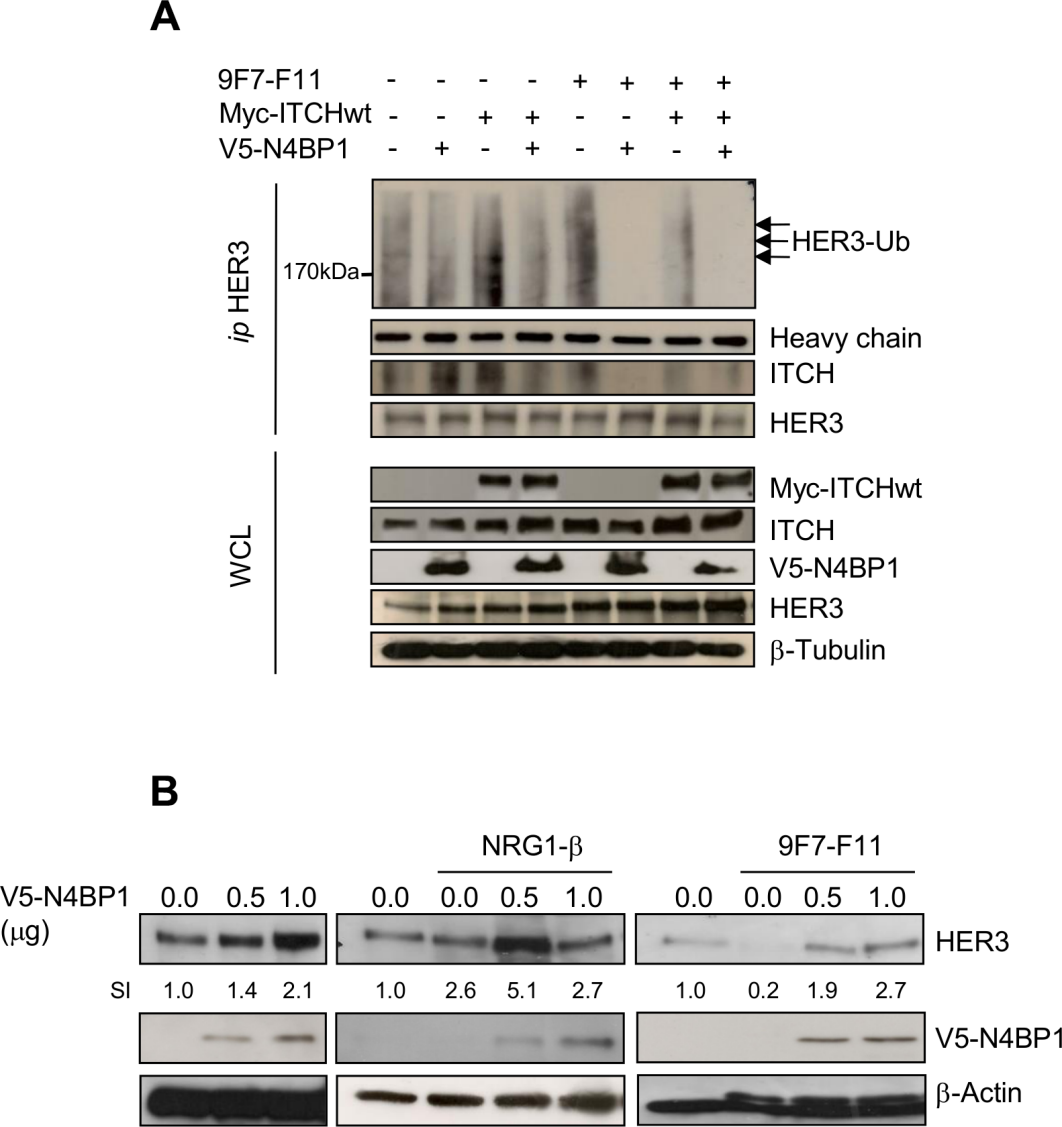
N4BP1 overexpression inhibits 9F7-F11-mediated HER3 ubiquitination and degradation induced by ITCH, and promotes HER3 protein stabilization in BxPc3 cells (**A**) Cells were co-transfected with the Myc-ITCHwt, HA-Ub and/or GFP-N4BP1 plasmids for 24 hr. Transfected cells were then incubated with 20 μM MG132 for 5 hr before addition of 50 μg/mL 9F7-F11 for 3 hr. After cells lysis with the CHAPS buffer and immunoprecipitation with the HER3 Ab, the ubiquitination status was analyzed by western blotting using an anti-HA antibody. HER3 and ITCH were detected using specific antibodies. (**B**) Cells were transfected with increasing doses of the V5-N4BP1 plasmid for 24 hr, and then incubated with 50 μg/mL 9F7-F11 or with 100 ng/mL NRG-1β for 3 hr. HER3 expression was assessed in whole cell lysates by western blotting. The V5-HRP antibody was used to detect N4BP1, and β-actin was the loading control. The signal intensity (SI) of the different bands was quantified with ImageJ. WCL, whole cell lysates.

We transiently overexpressed NEDD4 Binding Protein-1 (N4BP1; a specific ITCH inhibitor) [41] with HA-ubiquitin and wild type Myc-ITCH in BxPC3 (Figure 5) and MDA-MB468 (Supplementary Figure S5) cells, and tested HER3 ubiquitination by immunoprecipitation and western blotting. 9F7-F11-induced HER3 ubiquitination was abrogated by N4BP1 overexpression in BxPC3 cells (Figure 5A). Moreover, N4BP1 overexpression inhibited 9F7-F11-induced HER3/ITCH interaction (Figure 5A); such effect being also demonstrated with another anti-HER3 antibody in MDA-MB468 cells (Supplementary Figure S5). Transfection of wild type Myc-ITCH did not enhance 9F7-F11-induced HER3 ubiquitination, probably due to the fact that ITCH activity is tightly regulated by auto-ubiquitination leading to its degradation [40, 42, 43]. To better understand N4BP1 role in HER3 protein stability, we transfected increasing concentrations of the V5-N4BP1 plasmid in BxPC3 cells. N4BP1 overexpression enhanced HER3 expression in a dose-dependent manner, compared to control (mock) (Figure 5B) in NRG1-stimulated cells, suggesting that NRG1-induced degradation could be controlled by ITCH, as proposed by others [36, 44]. Similar results were obtained following incubation of N4BP1-overexpressing cells with 9F7-F11. Conversely, HER3 levels were reduced upon 9F7-F11 addition in control cells. These results demonstrate that ITCH inhibition by N4BP1 reduces 9F7-F11-induced HER3 degradation and strengthen the hypothesis that ITCH is the main driver of 9F7-F11-induced HER3 ubiquitination and degradation.

### ITCH silencing inhibits 9F7-F11-induced HER3 degradation/downstream signaling blockade and 9F7-F11-induced HER3 ubiquitination

**Figure 6:**
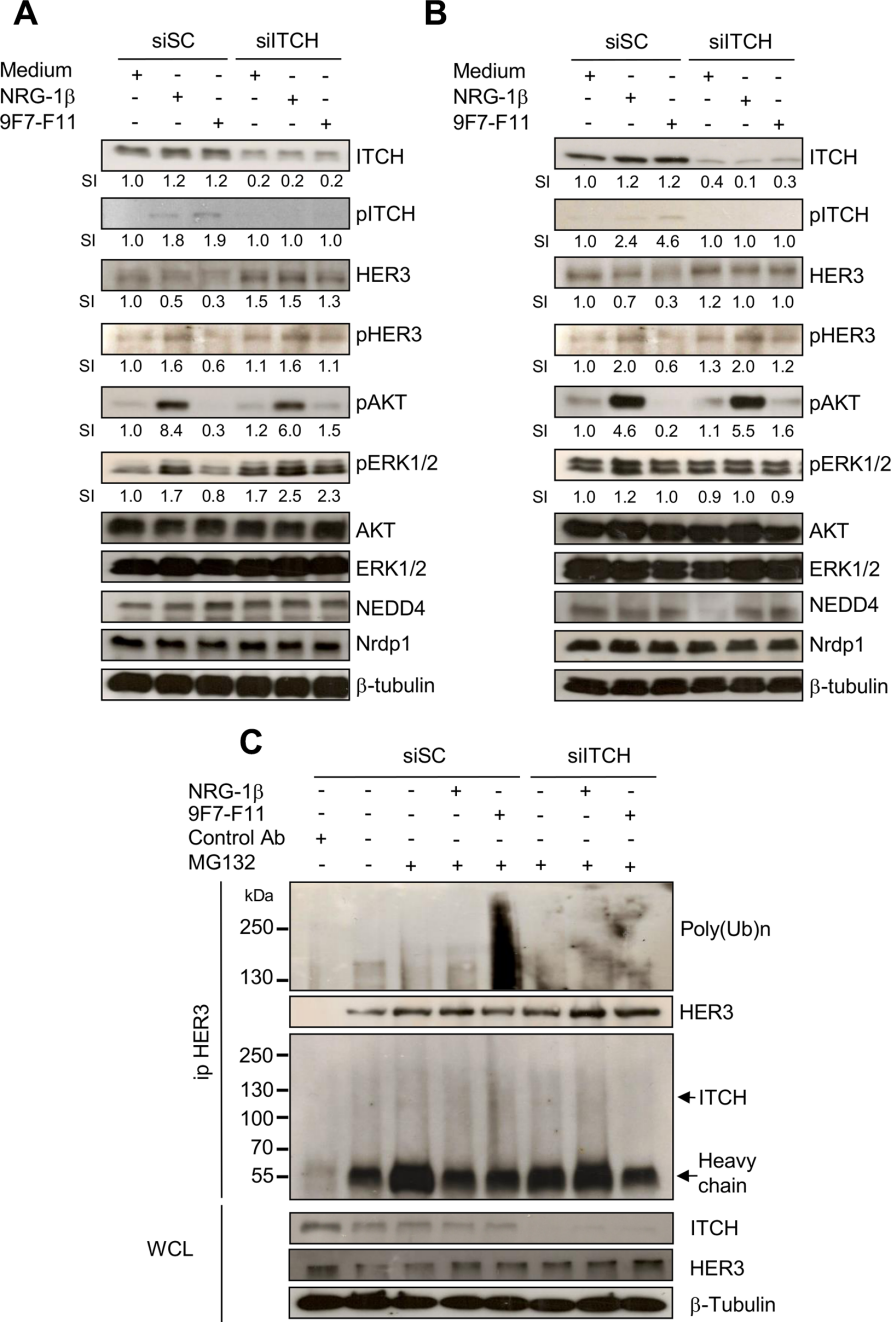
ITCH silencing inhibits 9F7-F11-mediated HER3 degradation and ubiquitination in cancer cells Pancreatic BxPC3 (**A**) and prostatic DU145 (**B**) cancer cells were transfected with 10 nM Scramble Control siRNA (siSC) or the anti-*ITCH/AIP4* siRNA (siITCH) for 72 hr, serum-starved and then incubated with 50 μg/mL 9F7-F11 or with 100 ng/mL NRG-1β for 4 hr. ITCH, HER3, AKT, ERK1/2, NEDD4 and Nrdp1 protein expression and ITCH, HER3, AKT and ERK1/2 phosphorylation were assessed in whole cell lysates (WCL) by western blotting. Band signal intensity (SI) was quantified with ImageJ, and β-tubulin was used as loading control. (**C**) BxPC3 cells were transfected with 10 nM siSC or siITCH for 72 hr, and then pre-incubated with 10 μM MG132 for 4 hr before addition of 9F7-F11 or NRG1-β for 4 hr. After immunoprecipitation with HER Ab, the HER3 ubiquitination status was analyzed by western blotting with a specific poly-ubiquitin chain antibody. HER3 and ITCH proteins were also detected by using specific antibodies.

We knocked-down the *ITCH* gene by transfecting BxPC3 (Figure 6A) and DU145 (Figure 6B) cells with a specific siRNA (siITCH) for 72 hr, before incubation with 9F7-F11 or NRG-1β. ITCH expression was reduced in siITCH-transfected cells, compared to siSC-transfected cells, in all conditions. In basal conditions (medium alone), siSC did not affect HER3 expression. 9F7-F11-induced reduction of HER3 expression, observed in siSC-transfected cells, was inhibited in siITCH-transfected BxPC3 (Figure 6A) and DU145 (Figure 6B) cells. This confirms ITCH role in 9F7-F11-induced HER3 degradation. Interestingly, *ITCH* silencing increased HER3 expression either in basal conditions or in 9F7-F11-treated BxPC3 cells, suggesting a more complex crosstalk between E3 ubiquitin ligases [18, 33], deubiquitinases [36, 45, 46] and regulating proteins [40, 42, 43]. Similar results were obtained when cells were stimulated with NRG-1β (Figure 6A and 6B). *ITCH* silencing also decreased 9F7-F11-triggered ITCH phosphorylation and inhibited 9F7-F11 blockade on HER3, AKT and ERK phosphorylation (Figure 6A and 6B). Nrdp1 and NEDD4 expression were not affected in siSC- and siITCH-transfected cells in all conditions (Figure 6A and 6B). Irrelevant control antibody Px did modify neither HER3 pathway nor ITCH and NEDD4 expression (Supplementary Figure S1). To confirm that NEDD4 is not involved in 9F7-F11-induced HER3 degradation, we silenced the *NEDD4* gene alone, or in combination with *ITCH*, in BxPC3 cells (Supplementary Figure S6). *NEDD4* silencing did not abrogate 9F7-F11-induced HER3 degradation and had no effect on the antibody-mediated blockade of HER3 downstream signaling. Conversely, concomitant *NEDD4* and *ITCH* silencing inhibited 9F7-F11-induced HER3 degradation and restored HER3 phosphorylation, leading to AKT and ERK phosphorylation (Supplementary Figure S6). These results demonstrate ITCH direct role in 9F7-F11-induced HER3 degradation. To confirm ITCH involvement in 9F7-F11-induced HER3 ubiquitination, we analyzed HER3 ubiquitination using an anti-poly-ubiquitin chain antibody after immunoprecipitation with an anti-HER3 antibody of extracts from *ITCH*-silenced BxPC3 cells incubated with 9F7-F11 or NRG-1β for 4 hr (Figure 6C). Irrelevant control antibody Px did not immunoprecipitate the HER3 receptor (Figure 6C). The strong HER3 poly-ubiquitination induced by 9F7-F11 in siSC-transfected cells was abrogated in *ITCH*-silenced cells. This result confirms the direct involvement of ITCH in HER3 ubiquitination upon incubation with 9F7-F11.

### The anti-HER3 antibody 9F7-F11 activates ITCH through JNK1/2 phosphorylation

**Figure 7:**
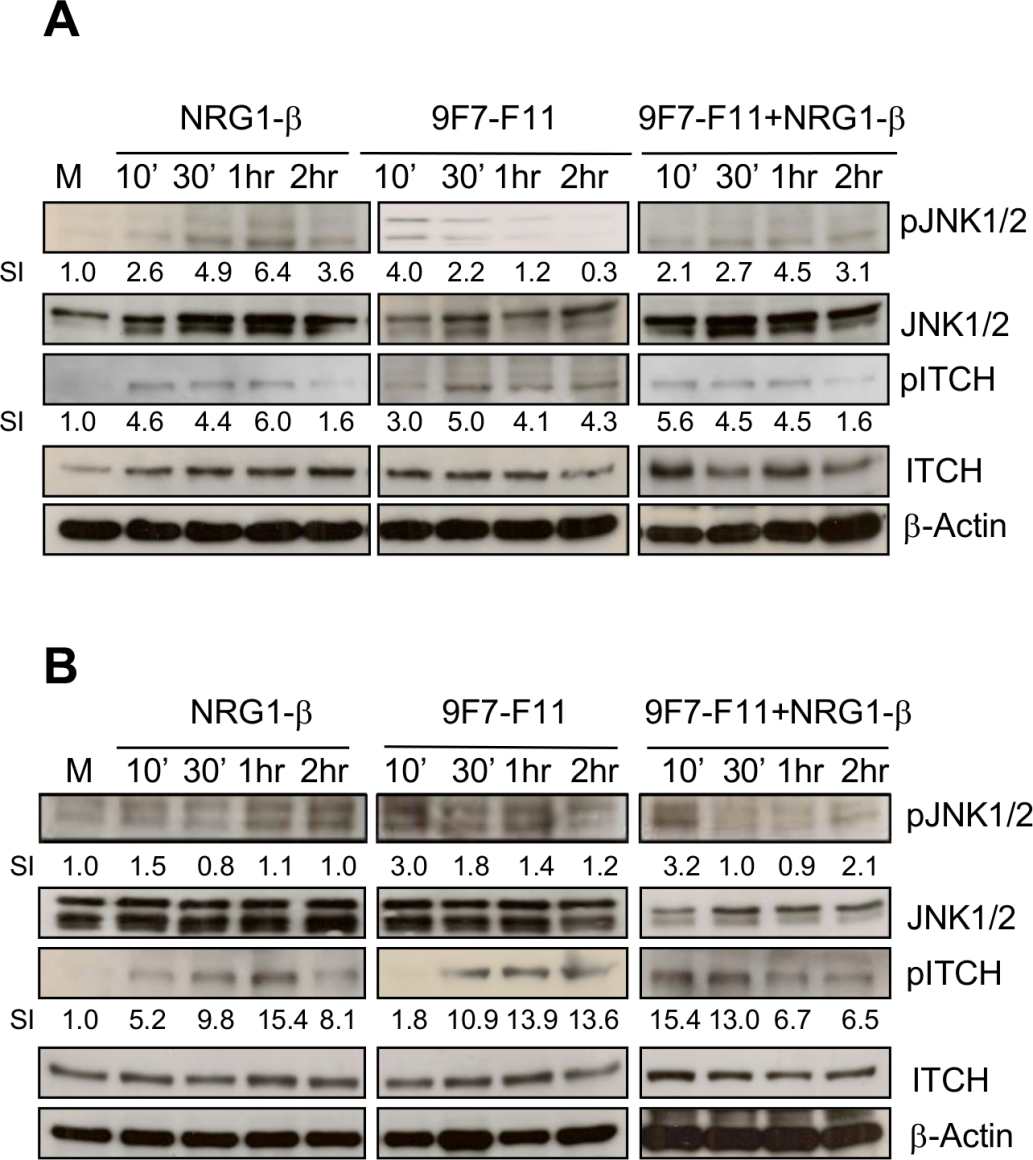
The anti-HER3 antibody 9F7-F11 induces JNK1/2 phosphorylation leading to ITCH activation in cancer cells Serum-starved cells from BxPC3 (**A**) and DU145 (**B**) cell lines were incubated with 100 ng/mL NRG-1β and/or 50 μg/mL 9F7-F11 for the indicated times. JNK1/2 and ITCH protein expression and their phosphorylation status were assessed in whole cell extracts by western blotting using the appropriate antibodies. Band signal intensity (SI) was quantified with ImageJ and β-actin was used as loading control.

Upon activation through JNK1/2-induced phosphorylation on Thr222, ITCH/AIP4 conformation changes from close to open, allowing substrate accessibility to its WW domains [39]. To determine whether JNK1/2 mediates ITCH activation during 9F7-F11-induced HER3 ubiquitination and degradation in cancer cells, we analyzed by western blotting JNK1/2 (at Thr183/Tyr185) and ITCH (at Thr222) phosphorylation in BxPC3 (Figure 7A), DU145 (Figure 7B) and MDA-MB468 (Supplementary Figure S7) cancer cells following incubation with 9F7-F11 or/and NRG-1β, or medium alone (control). Compared to control, in 9F7-F11-treated BxPC3 cells (Figure 7A), JNK1/2 phosphorylation was induced after 10min of incubation (SI = 4) and was followed by maximal and sustained ITCH phosphorylation on Thr222 (SI = 5) after 30 min of incubation and up to the end of the experiment (2 hr). This effect was stronger in DU145 cells (Figure 7B) than in BxPC3 cells (Figure 7A). JNK1/2 expression and phosphorylation were not modified by irrelevant antibody Px (Supplementary Figure S1). Similar results were obtained when cells were stimulated with NRG1-β. ITCH protein level progressively decreased, suggesting that following its activation, ITCH is degraded, in accordance with previous work demonstrating that activated ITCH auto-ubiquitinates through lysine linkage to induce poly-ubiquitination of its substrates [40]. Finally, JNK inhibitor SP600125 blocked 9F7-F11-mediated JNK1/2 and ITCH phosphorylation, together with HER3 degradation (Supplementary Figure S8), without modifying 9F7-F11-induced inhibition of HER3 signalling. JNK1/2 blockade by SP600125 in NRG1-β-stimulated cells also abrogated ITCH activation and HER3 degradation, and slightly favored NRG1-β-induced HER3 signalling.

### The anti-HER3 antibody 9F7-F11 induces USP8 and USP9X expression, leading to ITCH stabilization

**Figure 8:**
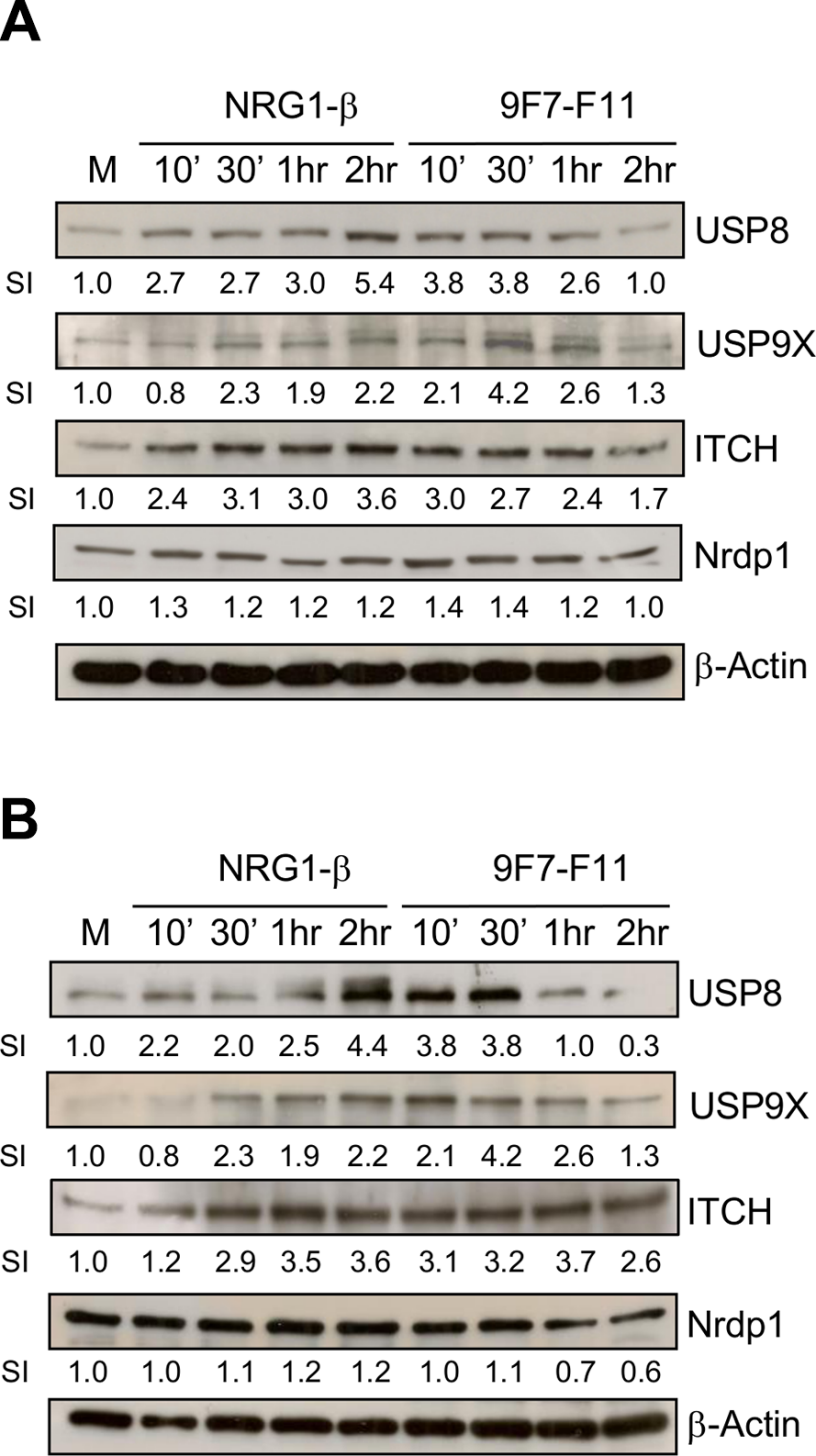
The anti-HER3 antibody 9F7-F11 increases USP8 and USP9X expression, leading to ITCH stabilization cancer cells Serum-starved cells from BxPC3 (**A**) and DU145 (**B**) cell lines were incubated with 100 ng/mL NRG-1β or 50 μg/mL 9F7- F11 for the indicated times. USP8, USP9X, ITCH and Nrdp1 protein expression were assessed in whole cell extracts by western blotting using the appropriate antibodies. Band signal intensity (SI) was quantified with ImageJ and β-actin was used as loading control.

USP8 and USP9X deubiquitinate ITCH to induce ubiquitination and degradation of the anti-apoptotic protein c-FLIP, leading to apoptosis in glioblastoma [45], or to anoikis in pancreatic ductal adenocarcinoma [46]. ITCH is stabilized upon interaction with USP9X [47]. To determine whether USP8 and/or USP9X stabilizes ITCH in 9F7-F11-treated cells, we analyzed USP8 and USP9X protein level in BxPC3 (Figure 8A) and DU145 (Figure 8B) cells. After 30 min incubation with 9F7- F11 in both cell lines, USP8 and USP9X expression was maximally increased by about 4-fold compared to cells with medium alone whereas control antibody Px did not modify expression (Supplementary Figure S1). The temporal kinetics of USP8/USP9X expression was comparable with that of ITCH in 9F7-F11-treated BxPC3 (Figure 8A) and DU145 (Figure 8B) cells. This suggests that USP8 and USP9X might be involved in controlling ITCH stability during 9F7-F11-induced HER3 ubiquitination and degradation. Similar results, but with a slower and longer USP8 and USP9X up-regulation, were obtained when BxPC3 or DU145 cells were stimulated by NRG-1β. In this case, USP8 expression was maximal after 2 hr of NRG-1β incubation, in close correlation with ITCH stabilization. The better correlation between ITCH and USP8 expression kinetics suggests that USP8 could be more efficient in stabilizing ITCH than USP9X. Nrdp1 activates NRG-1β-induced HER3 degradation [32] and USP8 prevents Nrdp1 auto-ubiquitination [48], with a strong correlation between USP8 expression and Nrdp1 stabilization [36]. However, we found that Nrdp1 was not up-regulated, demonstrating the absence of correlation between USP8 over-expression and Nrdp1 stabilization in 9F7-F11-treated BxPC3 (Figure 8A) and DU145 (Figure 8B) cells. This confirms that Nrdp1 is not involved in 9F7- F11-induced HER3 degradation.

### USP8 or USP9X silencing inhibits ITCH-mediated HER3 degradation induced by the anti-HER3 antibody 9F7-F11

**Figure 9:**
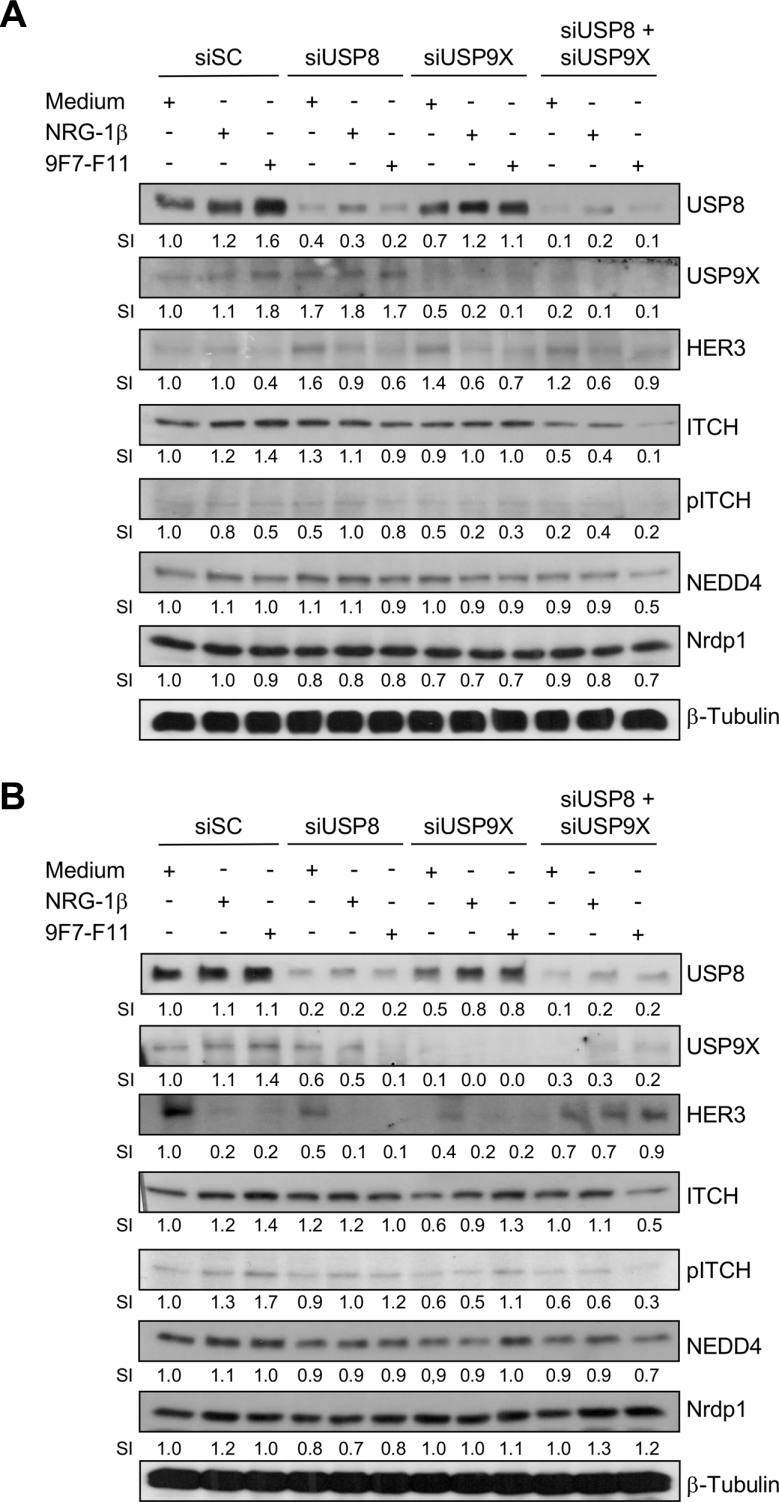
Simultaneous USP8/USP9X silencing inhibits ITCH-mediated HER3 degradation induced by 9F7-F11 in cancer cells BxPC3 (**A**) and DU145 (**B**) cancer cells were transfected with 10 nM scramble control siRNA (siSC), or anti-*USP8* (siUSP8) or/and anti-USP9X siRNA (siUSP9X) for 72 hr. Transfected cells were then serum-starved and incubated with 50 μg/mL 9F7-F11 antibody or with 100 ng/mL NRG1-β for 4 hr. USP8, USP9X, HER3, ITCH, NEDD4 and Nrdp1 expression were assessed in whole cell extracts by western blotting. ITCH phosphorylation at Thr222 was also evaluated using a specific pT222 antibody. β-tubulin was used as loading control. Band signal intensity (SI) was quantified with ImageJ.

It was previously reported that *USP9X* knock-down induces ITCH down-regulation in BxPC3 cells cultured in agar suspension, but not in monolayer [49]. To investigate USP9X and USP8 role in ITCH protein stabilization, we silenced the *USP8* or/and *USP9X* genes by siRNA transfection in BxPC3 (Figure 9A) and DU145 (Figure 9B) cells for 72 hr. *USP8* or *USP9X* silencing increased HER3 protein level in basal conditions (medium alone) in BxPC3 cells, suggesting that each deubiquitinase promotes basal HER3 degradation by stabilizing ITCH, as proposed for USP8 [36]. Conversely, ITCH expression was not modified, suggesting that when USP8 is down-regulated, USP9X maintains ITCH protein stability, and *vice versa*. Only concomitant silencing of USP8 and USP9X reduced ITCH expression, leading to loss of JNK-mediated ITCH phosphorylation on Thr222. Accordingly, 9F7-F11-induced HER3 degradation was inhibited in BxPC3 or DU145 cells in which both deubiquitinases were silenced. This suggests that USP8 and USP9X act concomitantly to favor ITCH stability, leading to 9F7-F11-induced HER3 ubiquitination and degradation. In contrast *USP8* and/or *USP9X* silencing did not modify 9F7-F11-induced inhibition of HER3 and AKT phosphorylation (Supplementary Figure S9).

## DISCUSSION

There are currently intense efforts towards the development of anti-HER3 antibodies for cancer treatment [8, 9, 50]. Most of these antibodies act by blocking the binding site for NRG-1β [10–12]. Targeting EGFR or HER2 by antibody-mediated ubiquitination and degradation [24, 25, 27–29, 51] is one attractive way to improve therapeutic benefit in patients. Such mechanism has been also proposed for antibodies against HER3 [15, 50, 52, 53] or IGFR1 [54]. Pre-clinical studies using anti-HER3 antibodies clearly identified receptor degradation as a marker of drug efficacy [55]. A mixture of anti-EGFR monoclonal antibodies that target different EGFR epitopes and increase EGFR ubiquitination and degradation in a CBL-independent manner led to improved efficacy of anti-EGFR therapy [24]. Finally, small molecules, such as the anti-anginal drug perhexilline, promote HER3 internalization and further ubiquitination, leading to inhibition of tumor growth [56].

In this study, we demonstrate that the therapeutic anti-HER3 antibody 9F7-F11 [15] induces HER3 ubiquitination and degradation through JNK1/2-dependent activation of the HECT E3 ubiquitin ligase ITCH/AIP4. This effect is co-activated by the deubiquitinases USP8 and USP9X. The AKT kinase has been previously shown to decrease USP8 function by increasing USP8 ubiquitination and decreasing active steady-state USP8 level [45]. 9F7-F11-induced inhibition of AKT phosphorylation [15] could relieve the blocking effect of AKT on USP8/USP9X function, allowing USP8/USP9X-mediated ITCH deubiquitination and stabilization. ITCH can induce ubiquitination of multiple proteins, including p63 and p73 [57, 58] and HER4 [22, 37], by interacting directly through its WW domains with a PPXY binding motif in the C-terminal tail of HER4 [22]. We identified this specific motif in the C-terminal tail of HER3 by sequence homology with HER4. This motif is also used for binding to NEDD4 [33], another HECT E3 ubiquitin ligase. Multiple E3 ubiquitin ligases can interact and regulate one substrate, depending on the cell context and the nature of the targeted protein. For example, in the EGFR family, HER4 is targeted by the two HECT E3 ubiquitin ligases WWP1 and ITCH to mediate its proteasomal and lysosomal degradation [21, 22]. Similarly, HER3 can be ubiquitinated by the RING E3 ubiquitin ligase Nrdp1 and also by NEDD4, leading to HER3 proteasome degradation [31–33]. In these cases, Nrdp1 and NEDD4 are only involved in HER3 degradation in basal conditions or after NRG-1β stimulation, but not in drug-induced HER3 degradation. This is in agreement with a previous report showing that NEDD4 participates in the basal ubiquitination and degradation of IGF-1R [59], but not following incubation with the specific antibody h7C10 [54]. Conversely, ITCH/AIP4 binds to and ubiquitinates HER3 upon incubation with the therapeutic antibody 9F7-F11, without affecting HER3 degradation in basal conditions. Nrdp1 interacts with HER3 juxtamembrane domain or kinase domain [60], whereas NEDD4 binds to the same motif [33] used by ITCH in HER3 C-terminal tail. The activity of these two ubiquitin ligases is probably regulated by the cell context (basal conditions *vs* drug-induced). This could explain why NEDD4 needs to be inhibited to sensitize cancer cells to anti-HER3 adjuvant therapy [33], whereas ITCH must be maintained to activate the drug biological effects, as we observed with the anti-HER3 antibody 9F7-F11. Therefore, compounds that indirectly increase ITCH expression, such as DNA methylase inhibitors that promote USP9X expression [46], could be beneficial.

Further studies are needed to understand first how 9F7-F11 induces JNK1/2 activation, leading to ITCH activation, and second how 9F7-F11 induces HER3 internalization and intracellular trafficking. 9F7-F11 antibody induces caspase-dependent cell apoptosis [15; unpublished results]. Oxidative stress is often associated with apoptosis and JNKs respond to cellular stress signals by phosphorylation. Therapeutic antibodies have been demonstrated to induce cell apoptosis together with reactive oxygen species accumulation, leading to JNK activation and phosphorylation [61, 62]. Thus we hypothesize that initiation of 9F7-F11-induced cell apoptosis could induce sustained stress generation, leading to JNK activation. ITCH is involved in protein trafficking by interacting with proteins of the ESCRT 0 complex, such as STAM and Hrs [63, 64], that could participate to antibody-induced HER3 degradation. The negative regulation of HER3 by 9F7-F11 via the ITCH E3 ligase has significant implications in our understanding of HER3 role in cancer biology, notably in pancreatic ductal adenocarcinoma. We demonstrate that the expression of the deubiquitinase USP9X, which activates ITCH stability, is increased in 9F7-F11-treated pancreatic cancer cells. Loss of USP9X expression leads to ITCH down-regulation and consequently could induce resistance to anoikis [46] and promote metastasis formation. Tumors with undetectable USP9X (13.6%) and ITCH (30.5%) have a worse prognosis [46]. ITCH down-regulation (and consequently reduction of HER3 ubiquitination and degradation) could maintain high HER3 expression in pancreatic ductal adenocarcinoma, driving metastasis formation *via* HER3 signaling. ITCH-mediated HER3 ubiquitination and degradation by 9F7-F11 could partly explain why this therapeutic antibody reduced tumor xenograft growth in nude mice [15, 65]. In addition, we can speculate that ITCH expression, and more broadly HER3 degradation, could be used as markers of response to anti-HER3 antibody targeted therapy, particularly in pancreatic and prostate cancer.

## MATERIALS AND METHODS

### Cells, antibodies and other reagents

The human pancreatic BxPC3, breast MDA-MB468 and prostate DU145 cancer cell lines were obtained from the American Type Culture collection (ATCC) (Rockville, MD). They all expressed the HER3 receptor (around 10,000 receptors/cell as quantified by flow cytometry) but not HER4. All cell lines were free of mycoplasma contamination, which was determined by the MycoAlert^™^ Detection Kit (Lonza, Switzerland). BxPC3 cells were cultured in Roswell Park Memorial Institute medium (RPMI) 1640. MDA-MB468 and DU145 cells were cultured in Dulbecco's modified Eagle's medium (DMEM). The culture media were supplemented as recommended by ATCC, usually with 10% fetal calf serum (FCS) and with penicillin and streptomycin (complete medium). All culture media and supplements were purchased from Life Technologies Inc. (Gibco BRL, Gaithersburg, MD). All cell lines were grown at 37°C in a humidified atmosphere with 5% CO_2_ and medium was replaced twice a week.

For western blotting, rabbit monoclonal antibodies against phospho-HER3 (Y1289), HER3, AKT, phospho-AKT (S473), ERK1/2 and phospho-ERK1/2 (Thr202/204), JNK1/2 and phospho-JNK1/2 (Thr183/Tyr185), USP9X, beta-actin and beta-tubulin were from Cell Signaling Technology (Danvers, MA). The mouse monoclonal antibody against ITCH (ref. 611199) was from BD Biosciences (San José, CA). The rabbit polyclonal antibodies against HER3 (C17), USP8 and USP9X were from Santa Cruz Biotechnology (Santa Cruz, CA). The rabbit polyclonal antibody against Nrdp1 was from Bethyl Laboratories (Montgomery, TX). The peroxidase-conjugated rabbit polyclonal anti-HA antibody, JNK inhibitor SP600125 (1,9-pyrazoloanthrone), MG132 and chloroquine were from Sigma-Aldrich (St Louis, MO). For detection of activated JNK and ITCH, we used a rabbit anti-phospho-JNK1/2 (Thr183/Tyr185) (clone 81E11) and an anti-phospho ITCH (Thr222) antibody (Millipore, Billerica, MA). We immunoprecipitated HER3 with the mouse monoclonal anti-HER3 antibody 2F12 (Millipore) against the epitope 1295–1323 in HER3 C-terminal domain. Human recombinant NRG-1β extracellular domain (ECD) was from RD Systems (Minneapolis, MN) and was used at 100 ng/mL. The normal control IgG antibody used for co-immunoprecipitation was obtained from Santa Cruz Biotechnology. The irrelevant control antibody Px, used for cell signaling experiments, is an IgG_1_ monoclonal antibody that was purified from the mouse myeloma cell line MOPC21. The mouse anti-HER3 therapeutic antibody 9F7-F11 was produced in our laboratory and used at 50 μg/mL [15].

### Transfection, overexpression and siRNA knockdown

For transient overexpression, the pcDNA-N4PB1-GFP vector (human full-length N4BP1 cDNA fused to GFP), the pcDNA-Myc-ITCHwt vector (full-length wild type ITCH cDNA fused to the Myc tag) and the pcDNA-HA-Ub vector (full-length linear ubiquitin fused to the HA tag) were generated by Gerry Melino. 2 × 10^6^ cells were plated in 10 cm-diameter dishes with complete RPMI or DMEM medium. When they reached 50–70% confluence, cells were transfected using Jet-PEI™ from Polyplus (New-York, NY) and 2 μg/plate of each vector for 24 hr. For siRNA-mediated knockdown, 2 × 10^6^ BxPC3 or DU145 cells were plated in 10 cm-dishes with RMPI medium without antibiotics until 50% confluence (about 24 hr). Cells were then transfected with 10 nM of pools of four specific different siRNAs against human *ITCH, Nrdp1, NEDD4, USP8, USP9X* respectively, or scramble control (ON-TARGETplus SMART pool, Dharmacon, Germany) in OptiMEM medium using Oligofectamine or Interferin^™^ (Life Technologies, Carlsbad, CA). After 4 hr of transfection, medium was replaced by RPMI for another 72 hr before using the cells for experiments.

### Cell lysis and immunoprecipitation

Cells were lysed in CHAPS buffer (Sigma-Aldrich) containing the protease inhibitor cocktail V (Calbiochem, Billerica, MA) and the phosphatase inhibitor cocktail II (Sigma-Aldrich). HER3 immunoprecipitation was performed by incubating 2 mg of cell lysate with 2 μg of the anti-HER3 antibody 2F12, which recognizes the HER3 intracellular C-terminal tail, at 4°C for 6 hr, followed by overnight incubation with 20 μl of protein A/G agarose beads (Santa Cruz Biotechnology) or with magnetic beads (Dynabeads^™^; Life Technologies) at 4°C under agitation. Samples were washed five times with 400 μl CHAPS buffer, re-suspended in 100 μl of 2X SDS Laemmli buffer and heated at 90°C for 10 min before electrophoresis. In each case, no HER3 protein was immunoprecipitated with beads alone or with normal control IgG antibody.

### Western blotting

2 × 10^6^ cells/dish were cultured at 37°C for 24 hr. After serum starvation in RPMI with antibiotics and 1% FCS for 24 hr, cells were incubated with various compounds. Treated cells were washed, scraped and lysed with CHAPS buffer (Sigma-Aldrich), as indicated above. After 5 hr of incubation at 4°C, the insoluble fraction was eliminated by centrifugation (13000 rpm, 10 min) and protein concentration in cell lysates was determined by the BCA assay. 200 μg of protein lysates were directly mixed with Laemmli buffer and heated at 95°C for 5min. After SDS-PAGE in reducing conditions, proteins were transferred to polyvinylidene difluoride membranes (Millipore) that were then saturated in TBS-Tween buffer (25 mM Tris pH 7.4, 150 mM NaCl, 0.1% Tween) containing 5% non-fat dry milk at 25°C for 1 hr. Membranes were then incubated with the appropriate dilution of primary antibodies in TBS-Tween/5% BSA buffer at 4°C for 18 hr. After two washes in TBS-Tween buffer, peroxidase-conjugated rabbit, goat or mouse polyclonal secondary antibodies (Sigma-Aldrich) were added, as appropriate, in TBS-Tween buffer containing 5% non-fat dry milk at 25°C for 1 hr. After three washes in TBS-Tween buffer, antibody binding was visualized using a chemiluminescent substrate (Western Lightning Plus-ECL, Perkin Elmer, Waltham, MA). Quantification was done using Image J. The signal intensity (SI) of each protein detected in non-treated cells served as the reference for estimating the expression changes (SI > 1 or SI < 1) of a given protein.

## SUPPLEMENTARY MATERIAL FIGURES


