# Correction: The anti-HER3 (ErbB3) therapeutic antibody 9F7-F11 induces HER3 ubiquitination and degradation in tumors through JNK1/2- dependent ITCH/AIP4 activation

**DOI:** 10.18632/oncotarget.28664

**Published:** 2024-10-11

**Authors:** Christophe Le Clorennec, Yassamine Lazrek, Olivier Dubreuil, Christel Larbouret, Marie-Alix Poul, Philippe Mondon, Gerry Melino, André Pèlegrin, Thierry Chardès

**Affiliations:** ^1^IRCM, Institut de Recherche en Cancérologie de Montpellier, Montpellier, F-34298, France; ^2^INSERM, U1194 Montpellier, Montpellier, F-34298, France; ^3^Université de Montpellier, Montpellier, F-34298, France; ^4^Department of Health and Endocrinology, University Magna Graecia of Catanzaro, Catanzaro, Italy; ^5^Millegen SA, Labège, F-31670, France; ^6^Biochemistry Laboratory, Instituto Dermopatico Dell’Immacolata, Department of Experimental Medicine and Surgery, University of Rome “Tor Vergata,” 00133 Rome, Italy; ^7^Toxicology Unit, Medical Research Council, Leicester University, Leicester LE1 9HN, United Kingdom; ^8^Institut Pasteur de Guyane, BP 6010, 97306, Cayenne Cedex, France; ^9^GamaMabs Pharma SA, Centre Pierre Potier, ONCOPOLE, BP 50624, France; ^10^LFB Biotechnologies, 59000, Lille, France

**This article has been corrected:** Due to errors during figure assembly, the pHER3 WB of BxPC3 cells in Figure 6A has been accidentally duplicated in the pHER3 line of Figure 6B. The corrected Figure 6, obtained using original data, is shown below. The authors declare that these corrections do not change the results or conclusions of this paper.

Original article: Oncotarget. 2016; 7:37013–37029. 37013-37029. https://doi.org/10.18632/oncotarget.9455

**Figure 6:**
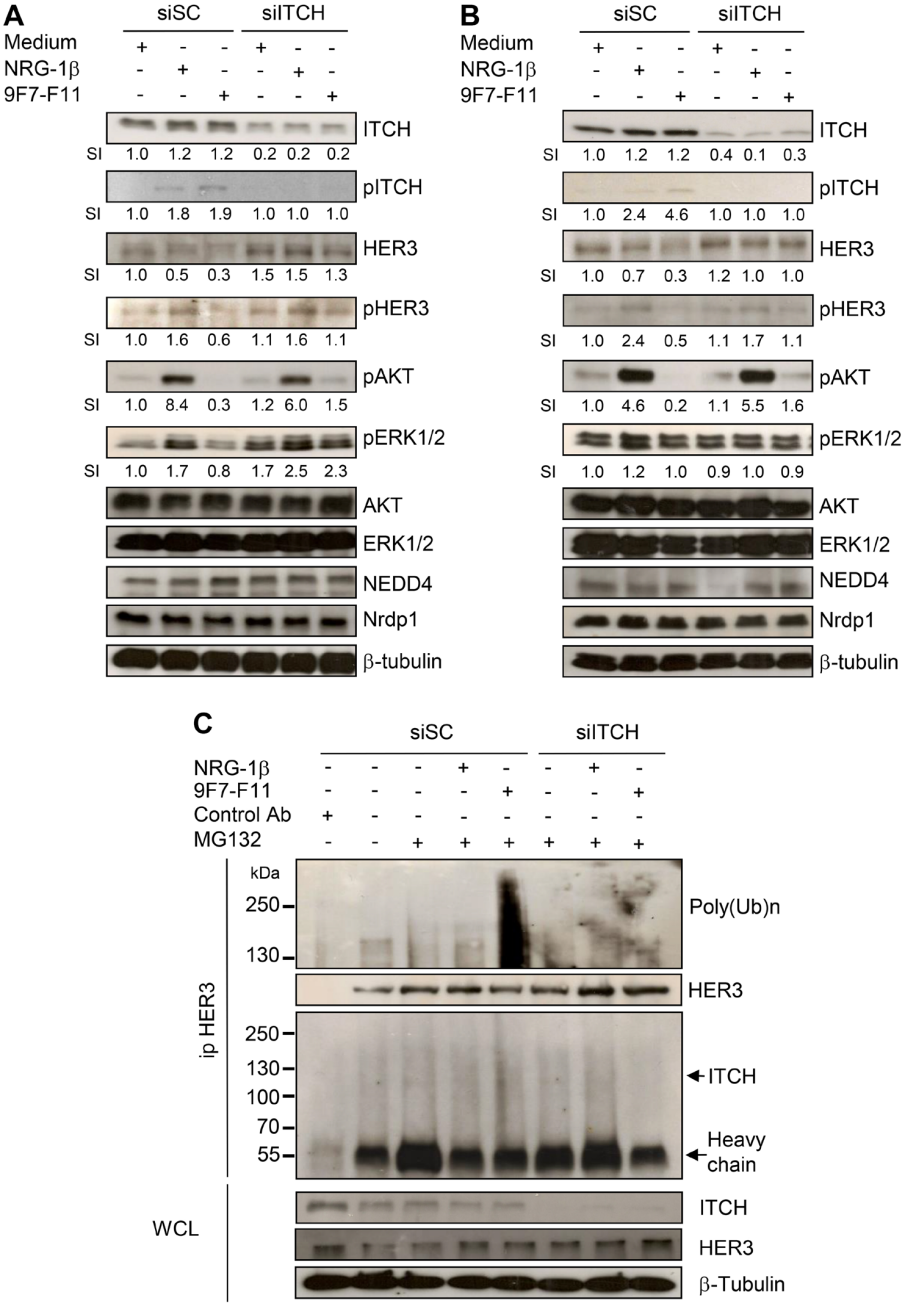
ITCH silencing inhibits 9F7-F11-mediated HER3 degradation and ubiquitination in cancer cells. Pancreatic BxPC3 (**A**) and prostatic DU145 (**B**) cancer cells were transfected with 10 nM Scramble Control siRNA (siSC) or the anti-*ITCH/AIP4* siRNA (siITCH) for 72 hr, serum-starved and then incubated with 50 μg/mL 9F7-F11 or with 100 ng/mL NRG-1β for 4 hr. ITCH, HER3, AKT, ERK1/2, NEDD4 and Nrdp1 protein expression and ITCH, HER3, AKT and ERK1/2 phosphorylation were assessed in whole cell lysates (WCL) by western blotting. Band signal intensity (SI) was quantified with ImageJ, and β-tubulin was used as loading control. (**C**) BxPC3 cells were transfected with 10 nM siSC or siITCH for 72 hr, and then pre-incubated with 10 μM MG132 for 4 hr before addition of 9F7-F11 or NRG1-β for 4 hr. After immunoprecipitation with HER Ab, the HER3 ubiquitination status was analyzed by western blotting with a specific poly-ubiquitin chain antibody. HER3 and ITCH proteins were also detected by using specific antibodies.

