# Supplementary material for: The anti-HER3 (ErbB3) therapeutic antibody 9F7-F11 induces HER3 ubiquitination and degradation in tumors through JNK1/2- dependent ITCH/AIP4 activation
Source: Oncotarget. 2016 May 18;7(24):37013–29. doi: 10.18632/oncotarget.9455 (PMC5095055; doi:10.18632/oncotarget.9455)
Supplement: Supplementary file 1 [file oncotarget-07-37013-s001.pdf]

The anti-HER3 (ErbB3) therapeutic antibody 9F7-F11 induces HER3 ubiquitination and degradation in tumors through JNK1/2-dependent ITCH/AIP4 activation

Supplementary Materials

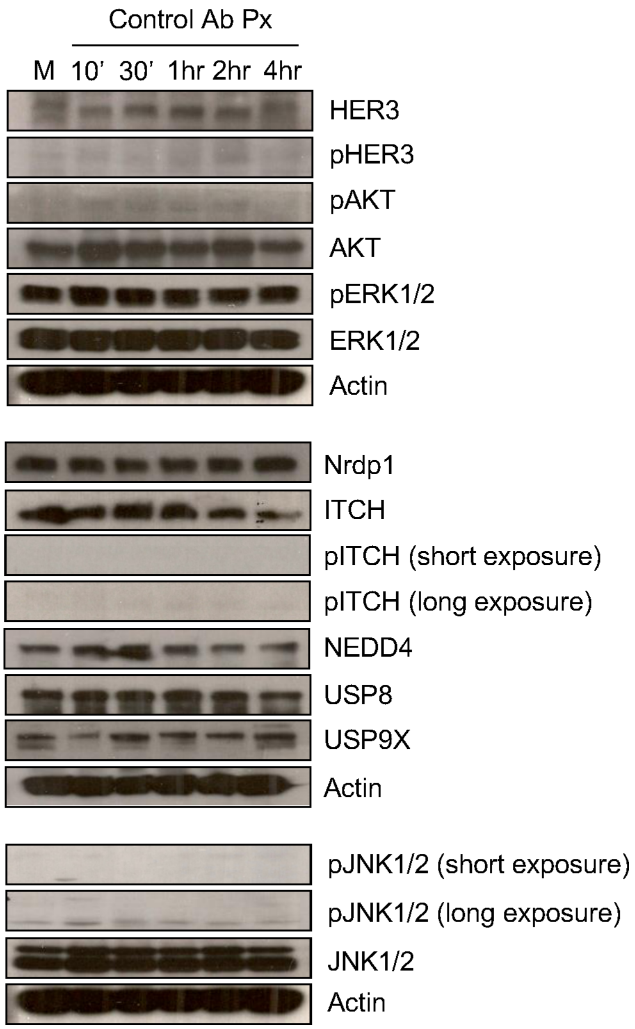

Supplementary Figure S1: The control antibody Px does not modify cell signalling. BxPC3 cancer cells were starved in 1% serum for 24 hr before treatment with 50 µg/mL of the control antibody Px for the indicated times, β-actin was evaluated as loading control.

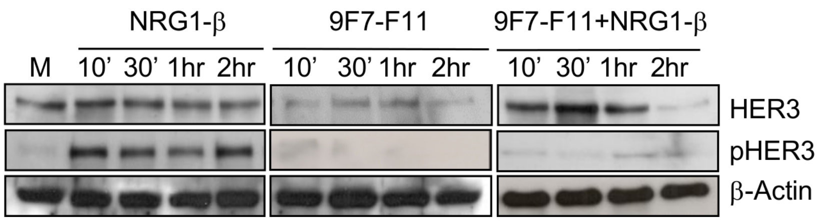

Supplementary Figure S2: The anti-HER3 antibody 9F7-F11 inhibits NRG1-β-induced HER3 phosphorylation and degradation in triple-negative breast cancer. MDA-MB468 cancer cells were starved in 1% serum for 24 hr before incubation with 100 ng/mL NRG-1β or/and with 50 μg/ml of the anti-HER3 antibody 9F7-F11 for the indicated times, β-actin was evaluated as loading control.

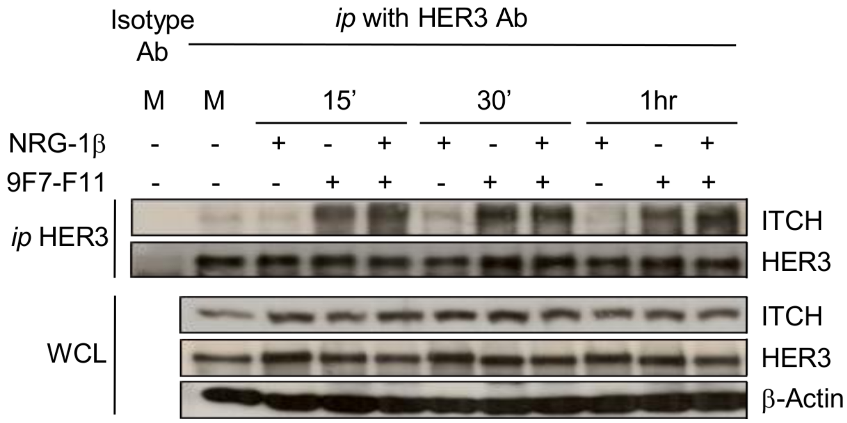

Supplementary Figure S3: 9F7-F11 induces ITCH recruitment to the HER3 receptor. Serum- starved BxPC3 cells were incubated with medium, 50 μg/mL 9F7-F11 or with 100 ng/ml NRG-1β for the indicated times. After immunoprecipitation (ip) of 2 mg of total protein extracts with an anti-HER3 monoclonal antibody against HER3 C- terminal tail (HER3 Ab) or an isotype control, ITCH and HER3 were detected by western blotting, β-actin was evaluated as loading control.

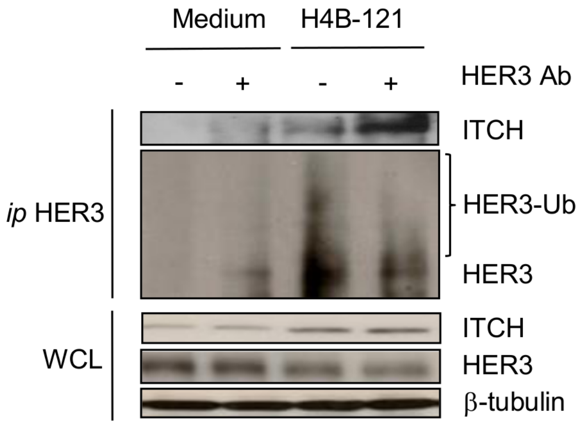

Supplementary Figure S4: The anti-HER3 antibody H4B-121 induces recruitment of ITCH for HER3 ubiquitination in MDA-MB468 breast cancer. MDA-MB468 cells were starved in 1% serum for 24 hr before incubation with 50 μg/ml of the anti-HER3 antibody H4B-121 during 1 hr. After immunoprecipitation (ip) of 2 mg of total protein extracts with an anti-HER3 monoclonal antibody against HER3 C-terminal tail (HER3 Ab), ITCH, HER3- Ubiquitin and HER3 were detected by western blotting, β-tubulin was evaluated as loading control. WCL, whole cell lysates.

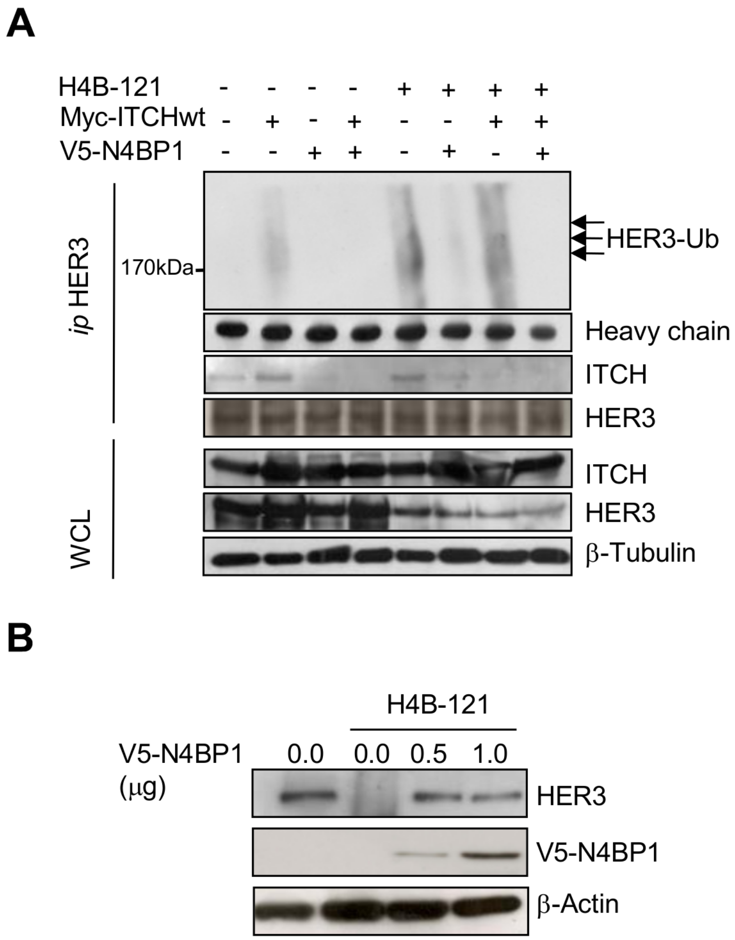

Supplementary Figure S5: N4BP1 overexpression inhibits H4B- 121-mediated HER3 ubiquitination and degradation induced by ITCH, and promotes HER3 protein stabilization in MDA-MB468 cells. (**A**) Breast cancer cells were co-transfected with the Myc-ITCHwt, HA-Ub and/or GFP-N4BP1 plasmids for 24 hr. Transfected cells were then incubated with 20 nM MG132 for 5 hr before addition of 50 µg/ml anti-HER3 antibody H4B-121 for 3 hr. After cells lysis with the CHAPS buffer and immunoprecipitation with the HER3 Ab, the ubiquitination status was analyzed by western blotting using an anti-HA antibody. HER3 and ITCH were detected using specific antibodies. (**B**) Cells were transfected with increasing doses of the V5-N4BP1 plasmid for 24 hr, and then incubated with 50 µg/ ml H4B-121 for 3 hr. HER3 expression was assessed in whole cell lysates by western blotting. The V5-HRP antibody was used to detect N4BP1, and β-actin was the loading control. WCL, whole cell lysates.

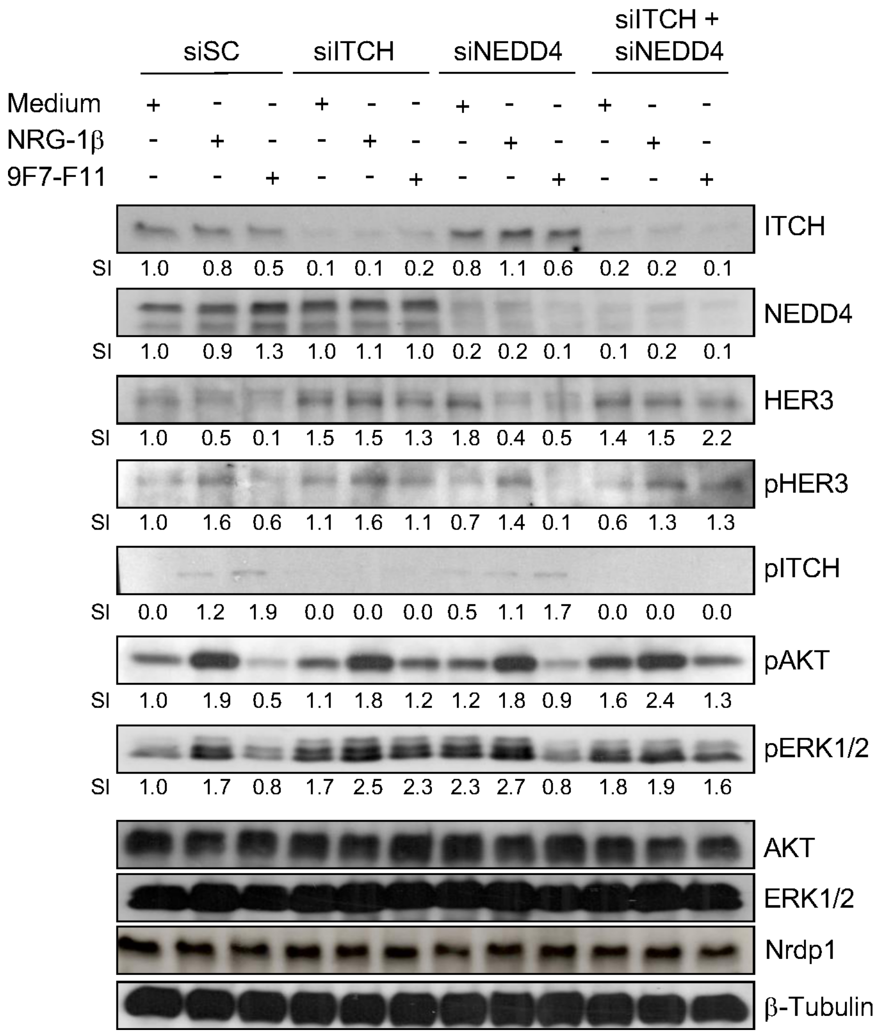

Supplementary Figure S6: NEDD4 silencing does not modify 9F7-F11 -induced HER3 degradation in BxPC3 pancreatic cancer cells. Cells were transfected with 10 nM Scramble Control siRNA (siSC) or the anti-*ITCH/AIP4* siRNA (silTCH) and/or the anti- NEDD4 siRNA (siNEDD4) for 72 hr. BxPC3 cells were further 18 h-starved in 1% serum and then incubated with 50 µg/ml 9F7-F11 or with 100 ng/ml NRG-1β for 4 hr. ITCH, NEDD4, HER3, AKT, ERK1/2 and Nrdpl protein expression, and HER3, ITCH, AKT and ERK1/2 phosphorylation were assessed in whole protein extracts by western blotting. Band signal intensity (SI) was quantified with ImageJ and β-tubulin was used as loading control.

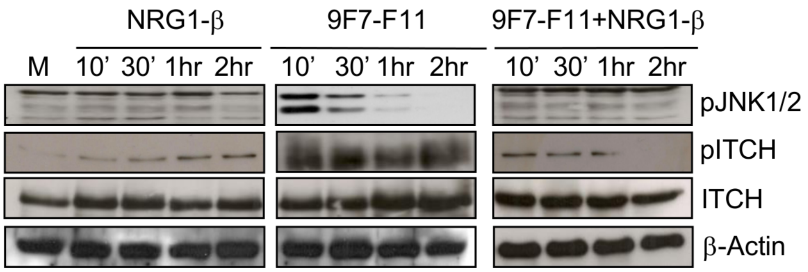

Supplementary Figure S7: The anti-HER3 antibody 9F7-F11 induces JNK1/2 phosphorylation leading to ITCH activation in MDA-MB468 breast cancer cells. Serum-starved cells from MDA-MB468 cell line were incubated with 100 ng/ml NRG-1β and/or 50 µg/ml 9F7-F11 for the indicated times. pJNK1/2, pITCH and ITCH were assessed in whole cell extracts by western blotting using the appropriate antibodies, β-actin was used as loading control.

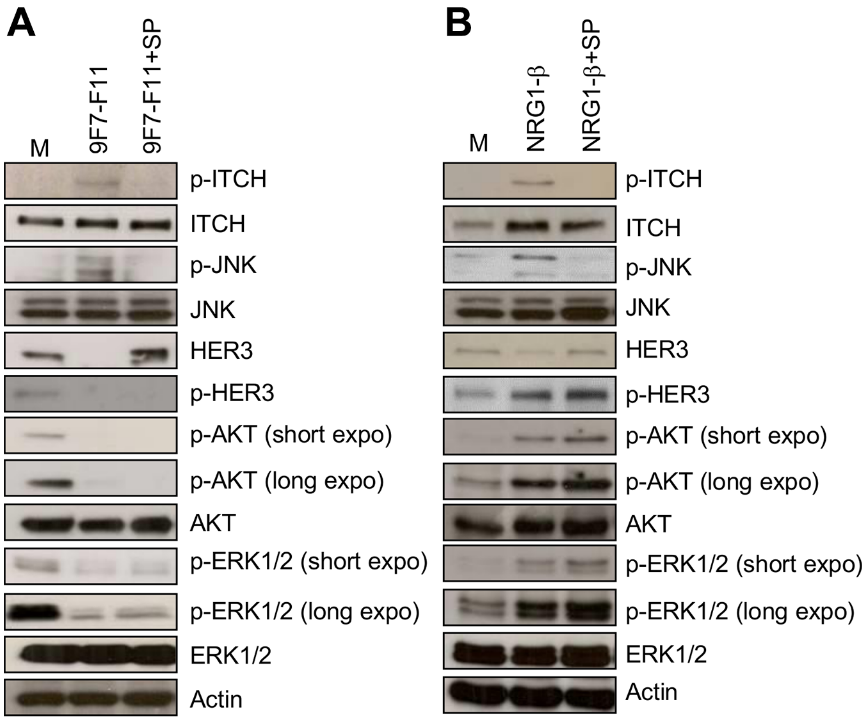

Supplementary Figure S8: Blockade of JNK1/2-dependent ITCH activation reverts 9F7-F11-induced HER3 degradation without modifying 9F7-F11-induced HER3 signalling inhibition. Serum-starved BxPC3 cells were pre-incubated with 10 μM of JNK inhibitor SP600125 during 1 hr before addition of 50 μ/ml 9F7-F11 (**A**) or 100 ng/ml of NRG-1β (**B**) for 4 hrs. JNK1/2, ITCH, HER3, AKT and ERK1/2 expression and phosphorylation were assessed in whole cell extracts by western blotting using the appropriate antibodies, p-actin was used as loading control.

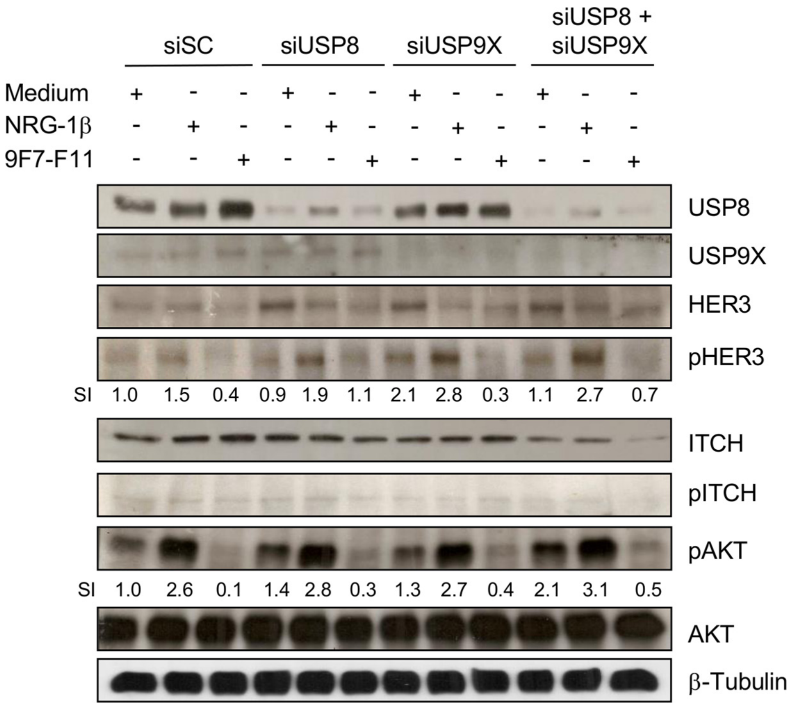

Supplementary Figure S9: Simultaneous USP8/USP9X silencing does not modify 9F7- F11-induced inhibition of HER3 and AKT phosphorylation. BxPC3 cancer cells were transfected with 10 nM scramble control siRNA (siSC), or anti-USP8 (siUSP8) or/and anti-USP9X siRNA (siUSP9X) for 72 hr. Transfected cells were then serum-starved and incubated with 50 μg/mL 9F7-F11 antibody or with 100 ng/mL NRG1-1β for 4 hrs. USP8, USP9X, HER3, ITCH and AKT expression were assessed in whole cell extracts by western blotting. HER3 (Tyr1289), ITCH (Thr222) and AKT (Ser473) phosphorylation were evaluated using appropriate antibodies, β-tubulin was used as loading control. Band signal intensity (SI) was quantified with ImageJ.
